# High prevalence of obstructive sleep apnea in a surgical aortic valve replacement cohort: an observational study

**DOI:** 10.1093/sleepadvances/zpae034

**Published:** 2024-05-30

**Authors:** Mark A Oldham, Wilfred R Pigeon, Michael Yurcheshen, Kazuhiro Hisamoto, Peter A Knight, Hochang B Lee

**Affiliations:** Department of Psychiatry, University of Rochester Medical Center, Rochester, NY, USA; Department of Psychiatry, University of Rochester Medical Center, Rochester, NY, USA; Center of Excellence for Suicide Prevention, U.S. Department of Veterans Affairs, Canandaigua, NY, USA; Department of Neurology, University of Rochester Medical Center, Rochester, NY, USA; Division of Cardiac Surgery, Department of Surgery, University of Rochester, Medical Center, Rochester, NY, USA; Division of Cardiac Surgery, Department of Surgery, University of Rochester, Medical Center, Rochester, NY, USA; Department of Psychiatry, University of Rochester Medical Center, Rochester, NY, USA

**Keywords:** sleep apnea, aortic valve disease, aortic stenosis, prevalence, aortic valve replacement

## Abstract

**Study Objectives:**

A high prevalence of sleep apnea has been reported among transcatheter aortic valve replacement (AVR) patients; however, the prevalence of sleep apnea in the younger and relatively healthier population of surgical AVR (SAVR) patients is unknown.

**Methods:**

We assessed the prevalence of sleep apnea and overall sleep quality in patients having SAVR. Participants aged 50–89 were eligible for recruitment. All participants completed type II HST before SAVR. Sleep apnea was defined as an apnea–hypopnea index (AHI) ≥ 5 events/hour. The current use of positive airway pressure was exclusionary.

**Results:**

The 46 participants (32 males/14 females) had a mean age of 66.6 years, body mass index of 30, AHI of 23.5, and obstructive AHI of 22.0. Only four participants had a prior sleep apnea diagnosis, yet all but one had sleep apnea on type II sleep testing. Two-thirds of sleep apnea was moderate or severe (AHI ≥ 15). A quarter of respiratory events were defined by arousals without desaturations. Whereas most sleep parameters resembled those of similarly aged community cohorts, mean percentage of N3 was reduced, accounting for only 3.8% of total sleep time.

**Conclusions:**

Type II home sleep testing (HST) revealed a 97.8% prevalence of sleep apnea in this sample, most of which was undiagnosed obstructive sleep apnea. Roughly two-thirds of sleep apnea was moderate or severe. Such a high impact of obstructive sleep apnea among patients with severe aortic valve disease deserves further investigation on potential underlying mechanisms and clinical implications.

**Statement of Significance**

All but one participant (45 of 46, 97.8%) in our sample of patients undergoing surgical aortic valve replacement (SAVR) had an apnea–hypopnea index of 5 or greater, only four of whom had a prior sleep apnea diagnosis. Two-thirds of our sample had moderate to severe sleep apnea. Most respiratory events were obstructive. To the best of our knowledge, this is the first study to characterize sleep apnea in a sample of patients undergoing SAVR. Our findings deserve replication and further research to explore potential explanations for such a high sleep apnea prevalence.

The prevalence of aortic valve disease (AVD) increases with age [1]. The most common type of AVD, aortic stenosis, occurs in 12.4% of older adults, with 3.4% of older adults having severe aortic stenosis [2]. The primary treatment for severe AVD is aortic valve replacement (AVR) [3], which may be surgical (surgical aortic valve replacement [SAVR]) or transcatheter (TAVR). SAVR is generally preferred for definitive treatment in patients with more favorable surgical risk profiles. As a result, the SAVR population has a lower baseline medical morbidity than the TAVR population and is roughly a decade younger than the population undergoing TAVR.

The prevalence of sleep apnea among SAVR populations is unknown. Studies have found a 62%–95% prevalence of sleep apnea in patients undergoing TAVR [4–7]; however, these results are of uncertain generalizability to those undergoing SAVR. Additionally, prior studies among patients with TAVR have utilized type III/IV home sleep testing (HST), which underestimates sleep apnea severity by failing to detect arousal-defined hypopneas and by using time in bed rather than total sleep time as the apnea–hypopnea index (AHI) denominator [8]. Sleep apnea can lead to cognitive impairment [9], which is especially relevant for patients having SAVR, as this population has an elevated risk of postoperative delirium [10] and subsequent cognitive impairment [11]. Sleep apnea could be one of the hidden mechanisms underlying the cognitive vulnerability of this population.

The American Academy of Sleep Medicine (AASM) recommends polysomnography (PSG) to diagnose sleep apnea in patients with major cardiorespiratory disease [12]. Whereas type I sleep tests (observed/in-lab PSG) are ideal, coordinating in-lab sleep tests before major surgery poses logistical challenges. Type II HST (unobserved PSG) offers greater feasibility and acceptability to patients while providing a more comprehensive evaluation than type III/IV HST. Furthermore, insurance companies do not generally consider AVD as qualifying comorbidity to reimburse for PSG; however, data establishing the high prevalence of sleep apnea in this population might encourage reconsideration of AVD as a qualifying condition. It might also lead to calls for routine screening for sleep apnea in this population, alongside other cardiovascular conditions including heart failure and recurrent atrial fibrillation [13].

Our primary aim is to describe the prevalence of sleep apnea and the distribution of its types and severity in a SAVR cohort. As secondary aims, we explore overall sleep parameters including sleep architecture (stages of sleep), sleep continuity (amount and distribution of wake and sleep periods across the night), and self-reported sleepiness and sleep quality.

## Materials and Methods

For this cross-sectional analysis of patients undergoing SAVR, we prospectively enrolled English-speaking participants ages 50–89 inclusive scheduled for SAVR at Strong Memorial Hospital. Participants were excluded for problematic substance use (> 1 on CAGE-Adapted to Included Drugs scale), prior heart surgery, concurrent surgical procedure (except coronary artery bypass grafting), infectious endocarditis, emergency surgery, adhesive allergy, or a chart or self-reported psychotic disorder. Also exclusionary was dementia, including chart or self-reported diagnosis, Telephone Interview for Cognitive Status score under 27, or Clinical Dementia Rating (CDR) global score above 0.5. Study sample participants using positive airway pressure (*n* = 11) are excluded from this analysis. The University of Rochester Medical Center Research Participants Review Board approved this research (feasibility #1921 and currently enrolling #6883 studies).

We obtained basic demographics, calculated Charlson comorbidity score, and scored CDR [14]. Participants completed Lawton instrumental activities of daily living (IADL) [15], Pittsburgh Sleep Quality Index (PSQI) [16], Epworth Sleepiness Scale (ESS) [17], and 9-item Patient Health Questionnaire (PHQ-9) [18]. Participants completed type II HST before surgery using Trackit^TM^ Sleepwalker^TM^ (Lifelines Ltd., UK), consisting of 10 leads (three electroencephalograms, two electrooculograms, three electromyograms, and two electrocardiograms), two plethysmography belts, nasal cannula, and finger oximetry (prior figure [19]). Sleep tests were reviewed manually for quality and scored using Esprit Nova client access software (Signalitica) per *AASM Manual v2.6*.

For the primary analysis, we reviewed the distribution of values for apnea–hypopnea index (AHI), obstructive AHI, and central AHI. We define sleep apnea as AHI ≥ 5, categorized as mild (5–14), moderate (15–29), or severe (≥ 30). One-way ANOVA is used to assess associations for continuous variables and chi-square for categorical variables. Finally, sleep parameters of participants with elevated AHI are reported, stratified by sleep apnea severity, to describe sleep architecture and variables of sleep continuity.

## Results

**Figure 1.**
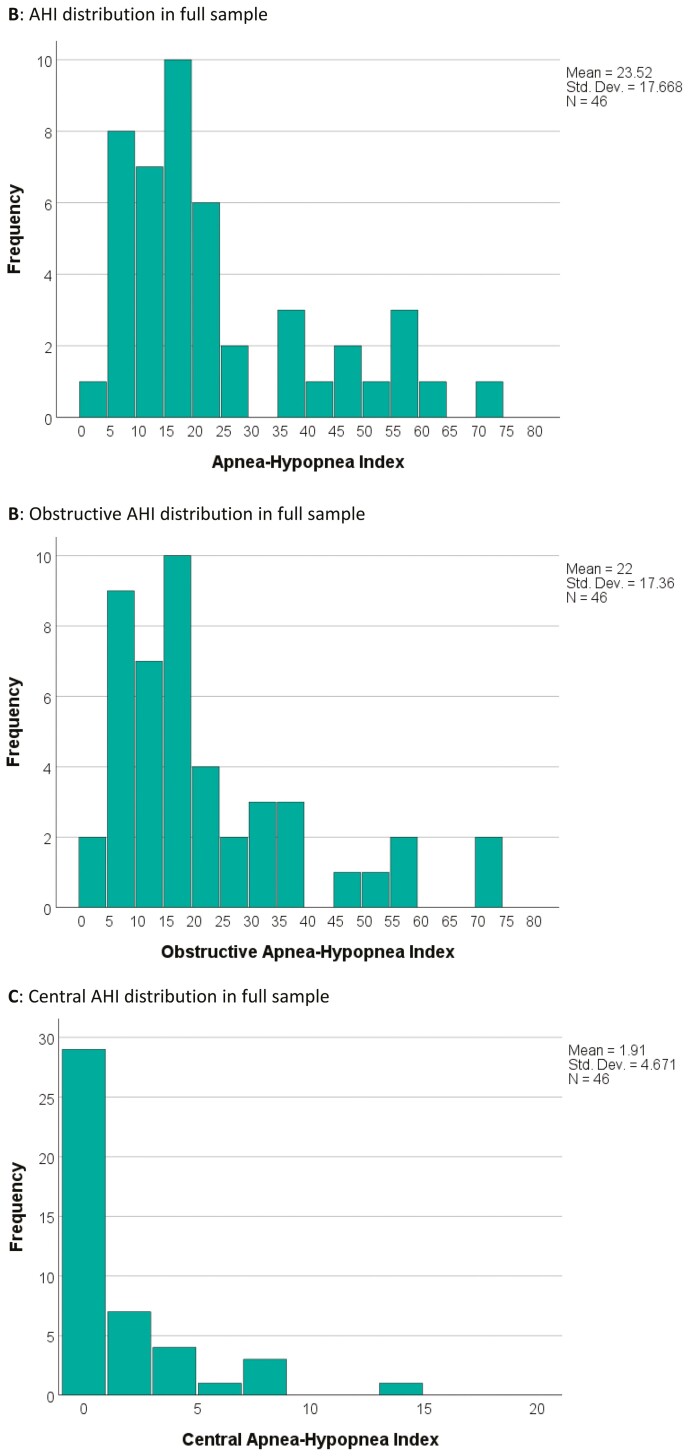
AHI distribution in full sample.

The study sample (*n* = 46) had a mean age of 66.6 years (± 8.5) and included 32 males (69.6%). Although only four (8.7%) participants had a prior sleep apnea diagnosis, 45 (97.8%) had sleep apnea on type II HST. The full sample mean AHI was 23.5 (Figure 1A). All but 2 of the 46 participants had an obstructive AHI of 5 or greater whereas six participants had a central AHI of 5 or greater. The mean obstructive AHI was 22.0 (± 17.4, Figure 1B) whereas the mean central AHI was 1.9 (± 4.7, Figure 1C). Three participants had incomplete oxygen sensor data. All events in these participants were scored using available data. Their AHIs were 13, 15, and 36, and their corresponding obstructive AHIs were 13, 15, and 34. As these values were elevated, they have been included in analyses throughout, though they may underestimate true values.

Table 1 describes sample characteristics excluding the participant with a normal-range AHI of 2. The distribution of mild, moderate, and severe elevations in AHI is relatively even: 15 participants had mild sleep apnea whereas 30 had moderate or severe sleep apnea. Most participants were white (93.3%), either married or living as married (71.1%), and had graduated college (64.4%). BMI ranged from 20 to 48. Although there was no statistical difference in mean BMI across the three severity levels, post hoc independent samples *t*-test comparing mild versus moderate-or-severe cases was statistically significant (27.4 ± 3.7 vs. 31.5 ± 6.5, two-sided *p* = .03, equality of variances not assumed). Similar post hoc independent samples *t*-tests and chi-square analyses of Table 1 variables revealed no differences between mild versus moderate or severe cases (data not shown). Six participants (13.3%) had mild cognitive impairment (i.e. global CDR score 0.5). IADL in this sample was broadly intact (max Lawton IADL score is 8). On questionnaires, 73% of participants reported impaired subjective sleep quality (PSQI ≥ 5); 15% reported elevated daytime sleepiness (ESS ≥ 11); and 11% reported moderate depression or greater (PHQ-9 ≥ 10). Scores on questionnaire did not vary by apnea severity category.

**Table 1.** Characteristics of Participants With Elevated AHI^1^

	Total (*n* = 45)	AHI 5–14 (mild) (*n* = 15)	AHI 15–29 (moderate) (*n* = 18)	AHI ≥ 30 (severe) (*n* = 12)	*P*-value^2^
Age, years	67.1 (7.7)	66.2 (9.8)	67.9 (7.3)	67.1 (5.3)	.83
Male	31 (68.9%)	10 (67%)	11 (61.1%)	10 (83.3%)	.43
Black	3 (6.7%)	1 (6.7%)	1 (5.6%)	1 (8.3%)	.57
Latinx	3 (6.7%)	1 (6.7%)	1 (5.6%)	1 (8.3%)	.57
Body mass index, kg/m^2^	30.1 (6.0)	27.4 (3.7)	32.4 (5.4)	30.07 (7.9)	.05
*Marital status*					.01
Married	29 (64.4%)	13 (86.7%)	8 (44.4%)	8 (66.7%)	
Living as married	3 (6.7%)	0	0	3 (25.0%)	
Divorced	5 (11.1%)	1 (6.7%)	4 (22.2%)	0	
Widowed	5 (11.1%)	0	4 (22.2%)	1 (8.3)	
Single	3 (6.7%)	1 (6.7%)	2 (11.1%)	0	
*Education*					.77
Grade school	1 (2.2%)	0	1 (5.6%)	0	
Some high school	1 (2.2%)	0	0	1 (8.3%)	
High school graduate	14 (31.1%)	4 (26.7%)	6 (33.3%)	4 (33.3%)	
College degree	20 (44.4%)	7 (46.7%)	8 (44.4%)	5 (41.7%)	
Post-graduate degree	9 (20.0%)	4 (26.7%)	3 (16.7%)	2 (16.7%)	
*Employment*					.66
Retired	22 (48.9%)	7 (46.7%)	10 (55.6%)	5 (41.7%)	
Full time	13 (28.9%)	5 (33.3%)	4 (22.2%)	4 (33.3%)	
Part-time	6 (13.3%)	3 (20%)	2 (11.1%)	1 (8.3%)	
Unemployed	1 (2.2%)	0	0	1 (8.3%)	
Disabled	3 (6.7%)	0	2 (11.1%)	1 (8.3%)	
*Medications* ^3^					
Beta-blocker	14 (31.1%	4 (26.7%)	7 (38.9%)	3 (25.0%)	.78
Benzodiazepine, scheduled	2 (4.4%)	0	2 (11.1%)	0	.33
Serotonin reuptake inhibitor	12 (26.7%)	5 (33.3%)	6 (33.3%)	1 (8.3%)	.23
*Charlson comorbidity index*	2.1 (2.0)	1.5 (1.4)	2.8 (2.6)	1.8 (1.3)	.15
Prior myocardial infarction	5 (11.1%)	1 (6.7%)	2 (11.1%)	2 (16.7%)	
Heart failure	5 (11.1%)	0	5 (27.8%)	0	
Peripheral vascular disease	1 (2.2%)	0	1 (5.6%)	0	
Prior CVA or TIA	3 (6.7%)	1 (6.7%)	2 (11.1%)	0	
Hemiplegia	0	0	0	0	
Pulmonary condition	12 (26.7%)	4 (26.7%)	5 (27.8%)	3 (25.0%)	
Diabetes, on treatment	8 (17.8%)	2 (13.3%)	4 (22.2%)	2 (16.7%)	
Diabetes, complication	5 (11.1%)	0	4 (22.2%)	1 (8.3%)	
Kidney disease	0	0	0	0	
Mild-moderate liver disease	1 (2.2%)	0	0	1 (8.3%)	
Severe liver disease	0	0	0	0	
Peptic ulcer disease	0	0	0	0	
Cancer	4 (8.9%)	1 (6.7%)	1 (5.6%)	2 (16.7%)	
Cancer, metastatic	0	0	0	0	
Dementia	0	0	0	0	
Connective tissue disease	0	0	0	0	
HIV disease	0	0	0	0	
Hypertension	30 (66.7%)	9 (60.0%)	15 (83.3%)	6 (50.0%)	
Skin ulcers/cellulitis	1 (2.2%)	0	1 (5.6%)	0	
Depression	9 (20.0%)	4 (26.7%)	4 (22.2%)	1 (8.3%)	
Coumadin	1 (2.2%)	0	1 (5.6%)	0	
Mild cognitive impairment	6 (13.3%)	2 (13.3%)	2 (11.1%)	2 (16.7%)	.91
Lawton IADLs	8.0 (0.2)	7.9 (0.3)	7.9 (0.2)	8.0 (0.0)	.69
*Self-report scales*					
Pittsburgh Sleep Quality Index	7.5 (3.5)	7.0 (2.8)	8.5 (3.6)	6.5 (4.1)	.26
Epworth Sleepiness Score	7.1 (4.2)	5.9 (3.5)	7.8 (5.1)	7.4 (3.4)	.42
Patient Health Questionnaire-9	5.2 (4.9)	5.1 (4.4)	5.5 (5.8)	4.7 (4.3)	.90
Average AHI, events/hr	24.0 (17.6)	9.33 (3.2)	19.0 (3.8)	49.8 (11.6)	<.001

^1^Excludes the one participant with AHI < 5.

^2^One-way ANOVA for continuous variables and chi-square for categorical variables.

^3^No participant was on a neuroleptic, and only 1 was on a scheduled opioid.

Abbreviations: AHI, apnea–hypopnea index; IADLs, instrumental activities of daily living.

Sleep parameters are shown in Table 2, again excluding the normal-range AHI participant. Whereas the mean obstructive AHI was 22.4, the desaturation index was only 17.0 indicating that respiratory arousals account for a non-negligible portion of hypopneas. Nearly half of recorded arousals were associated with respiratory events (9.0 of the 21.1/h). Regarding sleep architecture, mean proportion of N3 was reduced, accounting for only 3.8% of sleep. Sleep continuity variables revealed a total mean time in bed of 466.2 minutes. Sample means include a sleep latency of 33.6 minutes, wake after sleep onset of 62.9 minutes, total sleep time of 364.7 minutes (6.1 ± 1.2 h), sleep efficiency of 79.2%, and arousal index of 21.1/h. As expected, respiratory parameters, including respiratory arousal index, were increasingly elevated in association with greater sleep apnea severity; however, no associations between sleep stage times and sleep apnea severity were observed.

**Table 2.** Sleep Parameters of Participants With Elevated AHI

Sleep parameter	Mean (SD)	AHI 5-14 (mild) (*n* = 15)	AHI 15-29 (moderate) (*n* = 18)	AHI ≥ 30 (severe) (*n* = 12)	*P*-value^1^
Time in bed, min	466.2 (101.6)	456.6 (52.2)	449.7 (117.0)	502.9 (120.9)	.54
Total sleep time, min	364.7 (71.2)	365.2 (44.3)	351.8 (88.9)	383.4 (69.9)	.46
Sleep efficiency, %	79.2 (10.2)	80.3 (7.6)	78.9 (10.5)	78.2 (13.0)	.42
Sleep period time, min	432.9 (87.1)	432.1 (51.3)	411.6 (100.8)	465.8 (97.0)	.42
Wake after sleep onset, min	62.9 (45.1)	64.5 (31.8)	49.9 (39.5)	80.5 (61.9)	.21
Sleep latency, min	33.6 (33.1)	25.0 (17.1)	38.2 (37.3)	37.4 (41.5)	.56
REM latency, min	122.5 (81.2)	123.4 (92.8)	115.0 (71.3)	132.7 (85.9)	.86
*Respiratory parameters*					
Apnea–hypopnea index (AHI)^2^	24.0 (17.6)	9.3 (3.2)	19.0 (3.8)	49.8 (11.7)	<.001
Obstructive AHI^3^	22.4 (17.3)	8.9 (3.2)	17.5 (4.9)	46.8 (14.6)	<.001
Central AHI	2.0 (4.7)	0.4 (0.6)	0.7 (2.0)	5.8 (7.8)	.008
Desaturation index^4^	17.0 (14.0)	4.9 (3.7)	15.7 (5.9)	34.4 (13.6)	<.001
Mean O_2_ saturation, %^4^	92.5 (2.0)	93.7 (1.2)	91.5 (2.2)	92.6 (1.8)	.02
<90% O_2_ saturation, min^4^	23.6 (64.4)	1.8 (4.1)	41.0 (95.2)	24.5 (36.2)	.42
<90% O_2_ saturation, %^4^	6.2 (15.5)	0.5 (1.2)	10.8 (22.2)	6.5 (10.3)	.33
Snore index	60.1 (101.9)	21.3 (40.8)	97.4 (138.8)	52.9 (72.1)	.17
*Arousal*					
Arousal index	21.1 (9.1)	18.5 (7.1)	18.6 (5.6)	28.2 (12.0)	.01
Respiratory arousal index	9.0 (7.7)	3.9 (1.5)	7.1 (2.9)	18.3 (9.4)	<.001
Periodic limb movement arousal index	2.3 (3.5)	3.0 (5.5)	2.6 (2.1)	0.8 (0.9)	.31
Periodic limb movement index	21.2 (28.9)	21.2 (30.0)	30.3 (33.6)	7.8 (11.0)	.18
Isolated limb movement arousal total	1.6 (1.2)	2.0 (1.2)	1.7 (1.3)	0.9 (0.6)	.003
Isolated limb movement index	5.3 (3.2)	6.1 (3.4)	5.5 (3.2)	4.0 (2.8)	.26
Spontaneous arousal index	8.3 (3.9)	9.5 (4.7)	7.2 (2.9)	8.3 (3.9)	.39
*Sleep stages*					
N1, min	45.0 (20.8)	44.3 (16.4)	36.9 (21.1)	57.9 (20.5)	.05
N1, %	12.3 (5.6)	10.2 (3.4)	8.8 (4.3)	12.8 (5.2)	.13
N2, min	243.2 (53.9)	239.3 (32.7)	246.3 (71.5)	243.5 (48.2)	.59
N2, %	67.0 (9.6)	56.0 (10.5)	60.1 (12.6)	53.7 (12.2)	.36
N3, min	13.8 (18.9)	13.9 (19.3)	11.7 (19.8)	16.8 (18.2)	.88
N3, %	3.8 (5.2)	3.1 (4.3)	3.1 (5.2)	2.9 (3.1)	.98
REM, min	63.4 (32.7)	68.5 (32.9)	57.6 (28.1)	66.0 (39.8)	.75
REM, %	16.9 (7.3)	15.4 (6.6)	13.6 (5.6)	14.0 (8.2)	.85

^1^One-way ANOVA for continuous variables and chi-square for categorical variables.

^2^Excluding the three participants with incomplete oxygen sensor data, mean (SD) is 24.2 (18.0).

^3^Excluding the three participants with incomplete oxygen sensor data, mean (SD) is 22.6 (17.7).

^4^The three participants for whom these values are unreliable have been omitted from these parameters.

## Discussion

In this study sample, which omitted 11 participants on positive airway pressure, 45 of the 46 participants (97.8%) had an AHI of 5 or greater, with an even distribution across severity from mild to severe. Roughly two-thirds of this sample met the criteria for at least moderate sleep apnea. To the best of our knowledge, this is the first report to characterize sleep in a SAVR population.

This is also the highest prevalence of sleep apnea reported among patients with severe AVD, despite this sample’s lower mean age than previously reported TAVR samples. Prior reports have found 71% prevalence (mean age 72 [7] or 81 years [6]), 77% prevalence (81 years [5]), or 94% prevalence (83 years [4]). Our use of the more comprehensive type II HST could account for a portion of this higher prevalence as roughly a quarter of scored respiratory events were defined by arousals without corresponding desaturations. Furthermore, general population studies using type I or II sleep testing among older adults have reported sleep apnea rates approaching 90%, especially among men [20], though whether there could be a mechanistic link between sleep apnea and AVD is unclear.

The events in this sample were overwhelmingly obstructive, with only six participants (13%) having a central AHI of at least 5. This is consistent with one prior report [5] but differs from other TAVR cohorts, which have found higher rates of central sleep apnea ranging from 32% to 46% [4, 6, 7]. At close to 30, the mean BMI of this current sample is moderately higher than previous TAVR studies, with mean BMIs from 24 to 26 [4–7]. In one previous TAVR study, BMI did not differ between mild versus moderate or severe obstructive sleep apnea [5], although a similar analysis performed post hoc in this current study did find a statistical difference when similarly dichotomizing the sample. One also wonders whether the lower prevalence of central respiratory events in this study, versus prior TAVR reports, could be accounted for by the advanced age or greater degree of frailty in prior studies of TAVR cohorts.

Whereas PSQI indicated that three-quarters of participants had poor subjective sleep quality, only a quarter of participants reported elevated sleepiness on ESS. Importantly, the absence of relationship between ESS score and AHI in our sample questions the reliability of self-reported sleepiness as an index of sleep apnea severity in this population. Only modest depressive symptoms were reported in this sample.

Mean sleep continuity values suggested impaired sleep although, except for sleep latency, the level of impairment was broadly consistent with a first night of PSG among healthy older adults in this age range [21]. The prolonged REM latency could be related to a first-night effect with this device [22]. There was, however, a shift to lighter sleep stages. Comparing the mean percentage of time in each sleep stage of this current sample with healthy older adults [21], N3 percentage is markedly reduced (3.8% vs. 19.9%) and N2 percentage increased (67% vs. 53.3%). However, N1 and REM percentages are comparable to values of similarly aged older adults (12.3% vs. 9.3% and 16.9% vs. 17.7%, respectively).

Limitations to this current analysis include the absence of clinical assessment to determine a diagnosis of mild sleep apnea disorder in participants with an AHI from 5 to 14. Additionally, the HSTs performed in this study were unobserved, thereby introducing a potential source of error in HST channels. Nevertheless, data collection was nearly complete; recordings were of good quality on manual review; and the amount and type of data obtained from these type II HST exceeds that obtained from type III/IV HST. The oxygen saturation data from three participants are incomplete, which could lead to an artificially reduced AHI in these three participants. Although our gender and minority distribution is nearly identical to the study-eligible SAVR population at the institution from which our sample was recruited and resembles the national SAVR population [23], the fact that our sample was predominantly white and male limits generalizability to other demographics. Finally, we have not presented adjusted analyses because of the limited power afforded by this sample size. Our findings deserve replication and call for larger samples that allow for adjusted analyses to explain the high prevalence of sleep apnea in this population.

## Conclusions

Sleep apnea appears to be highly prevalent in the SAVR population. Most recorded events were obstructive in nature, which could be explained in part by sample demographics. Self-reported sleepiness was not a reliable index of AHI in this sample. Furthermore, the percentage of N3 sleep was significantly reduced and N2 increased. Several other objective sleep parameters and self-reported sleep quality reflect that the sample was sleep impaired, though much of this impairment appears to be in line with healthy older adults of similar age. The clinical implications of such a high impact of sleep apnea among patients with severe AVD, especially those undergoing SAVR, deserve further investigation.

## Data Availability

The data underlying this article will be shared on reasonable request to the corresponding author.
